# Supplementary material for: Morphological and Electrochemical Properties of ZnMn2O4 Nanopowders and Their Aggregated Microspheres Prepared by Simple Spray Drying Process
Source: Nanomaterials (Basel). 2022 Feb 18;12(4):680. doi: 10.3390/nano12040680 (PMC8880530; doi:10.3390/nano12040680)
Supplement: Supplementary file 1 [file nanomaterials-12-00680-s001.zip › nanomaterials-1585919-supplementary.pdf]

Supplementary Materials

Morphological and Electrochemical Properties of $ZnMn_2O_4$ Nanopowders and Their Aggregated Microspheres Prepared by Simple Spray Drying Process

Gi Dae Park [1], Yun Chan Kang [2,*] and Jung Sang Cho [3,*]

[1] Department of Advanced Materials Engineering, Chungbuk National University, Cheongju, 361-763, Korea; gdpark@cbnu.ac.kr

[2] Department of Materials Science and Engineering, Korea University, Anam-Dong, Seongbuk-Gu, Seoul 136-713, Korea

[3] Department of Engineering Chemistry, Chungbuk National University, Cheongju, 361-763, Korea

* Correspondence: yckang@korea.ac.kr (Y.C.K.); jscho@cbnu.ac.kr (J.S.C.); Tel.: +82-43-261-2489 (J.S.C.); Fax: +82-43-262-2380 (J.S.C.)

Electrochemical Measurements

1.1. Characterization Techniques

The crystal structure of the as-prepared and post-annealed powders was investigated by using X-ray diffraction (XRD, X'pert PRO MPD) with Cu K_a radiation (= 1.5418 Å), at the Korea Basic Science Institute (Daegu). The morphological characteristics of the prepared samples were investigated by using scanning electron microscopy (SEM, (S-4800, Hitachi, Tokyo, Japan) and a high-resolution transmission electron microscopy (TEM, JEM-2100F, JEOL, Tokyo, Japan). The specific surface areas of the aggregated microsphere powders were calculated from a Brunauer–Emmett–Teller (BET) analysis of nitrogen adsorption measurements (TriStar 3000).

1.2. Electrochemical Measurements

The electrochemical properties, including capacities and cycling performance of the powders, were determined by assembling a 2032-type coin cell. The anode was prepared by mixing 70 wt% of active material, 20 wt% of Super P® carbon black (H30253, Alfa Aesar, Haverhill, MA, USA) as the conductive material, and 10 wt% of sodium carboxymethyl cellulose (CMC, C5013, Sigma Aldrich, Saint Louis, MO, USA) as the binder. Lithium metal (010769, Alfa Aesar, Haverhill, MA, USA)and microporous polypropylene film were used as the counter electrode and separator, respectively. $LiPF_6$ (1 M) in a mixture of ethylene carbonate (EC) and dimethyl carbonate (DMC) in the volume ratio of 1:1 with 2 wt% vinylene carbonate (VC) was used as the electrolyte (Dongwha Electrolyte, Nonsan, Korea). The charge/discharge characteristics of the samples were determined by cycling in the voltage range 0.001–3.0 V. Cyclic voltammetry measurements were carried out at a scan rate of 0.07 mV s^{-1}.

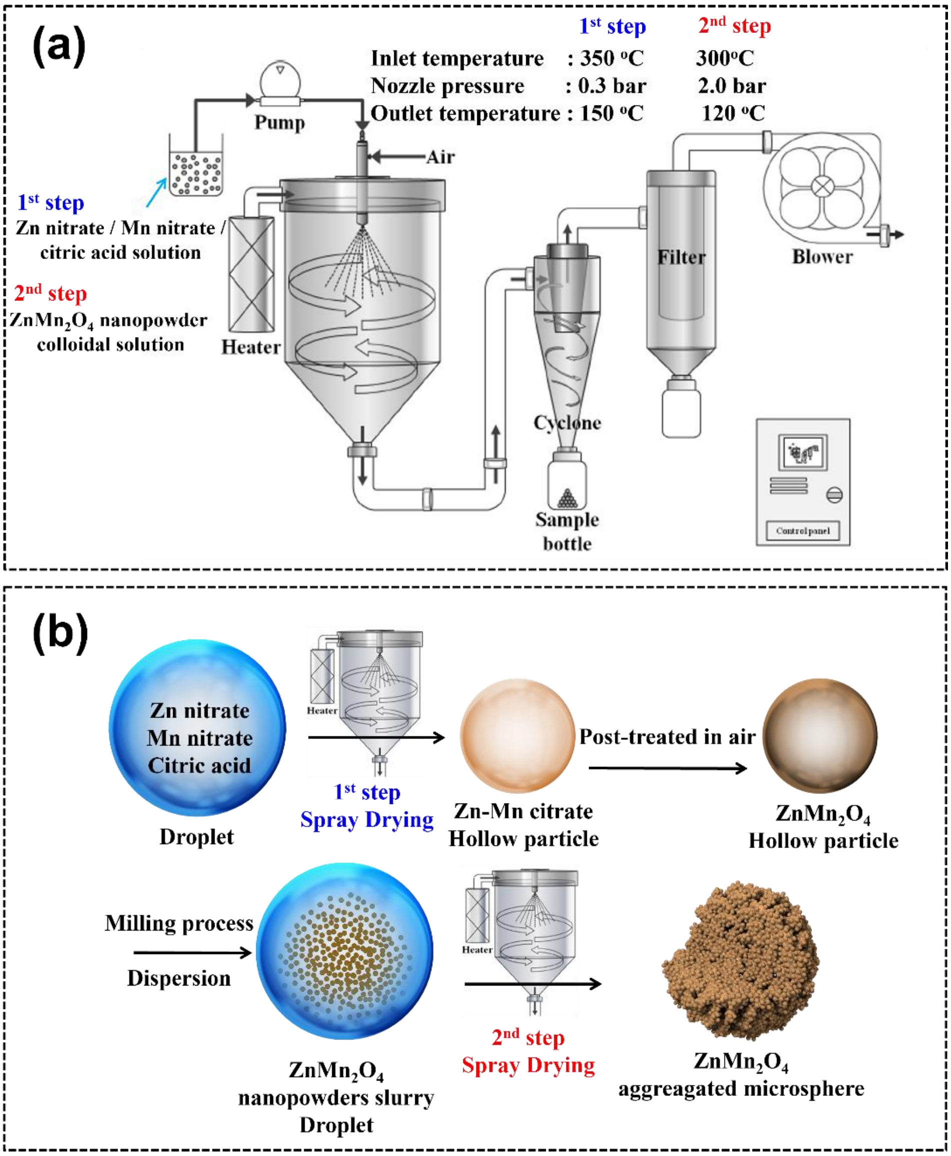

Figure S1. (**a**) Schematic diagram of spray drying system and (**b**) formation of $ZnMn_2O_4$ aggregated microsphere by 2 step spray drying process.

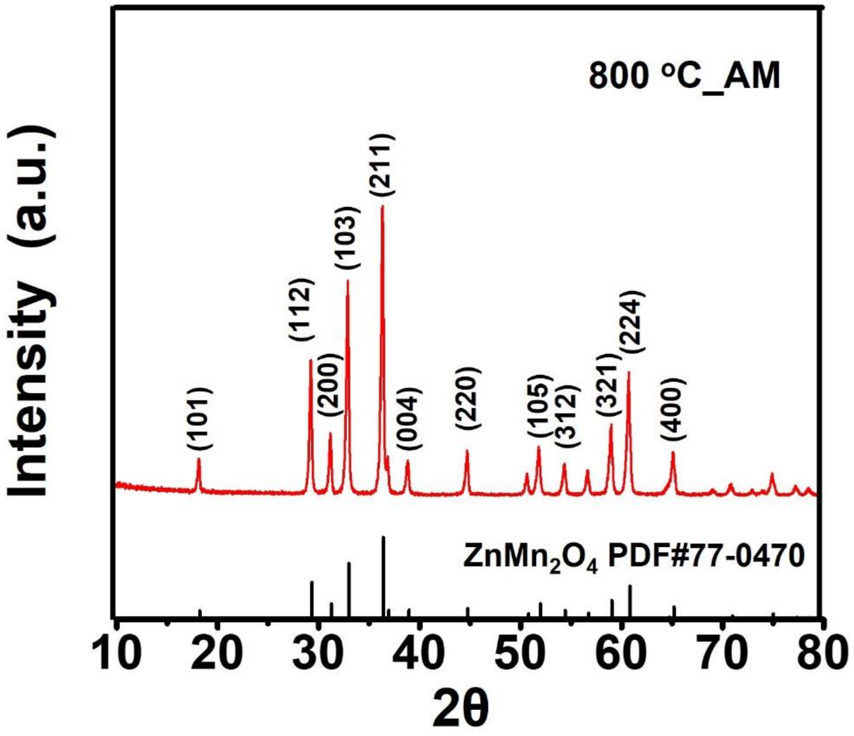

Figure S2. XRD pattern of the aggregated microsphere consisting of $ZnMn_2O_4$ nanoparticles, which were prepared at 800 °C post-treatment temperature.

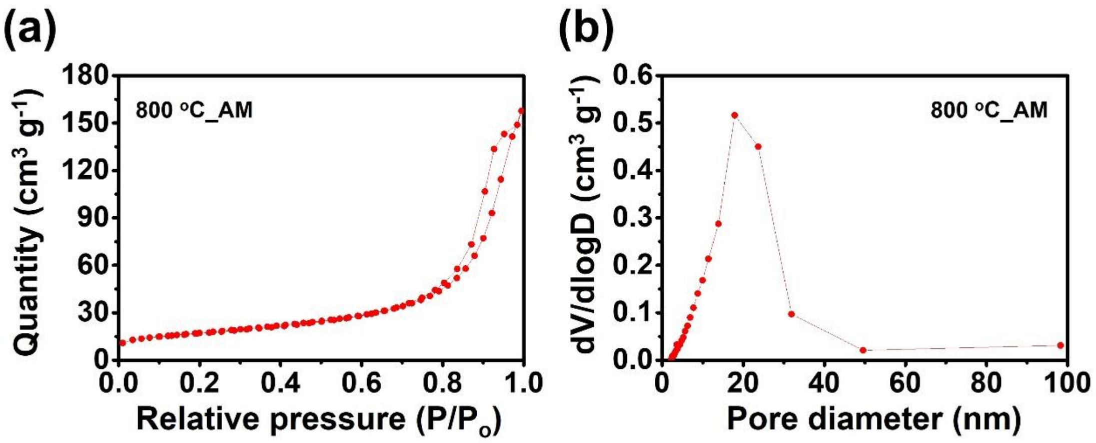

Figure S3. (**a**) N_2 gas adsorption and desorption isotherm and (**b**) BJH pore-size distribution of the aggregated microsphere consisting of $ZnMn_2O_4$ nanoparticles, which were prepared at 800 °C post-treatment temperature.

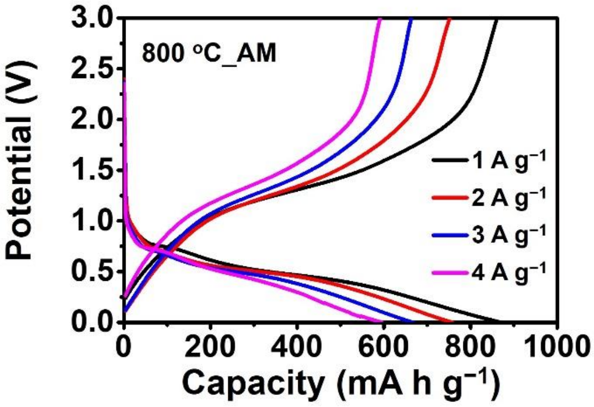

Figure S4. The charge and discharge profiles of $ZnMn_2O_4$ 800 °C_AM at different current density.

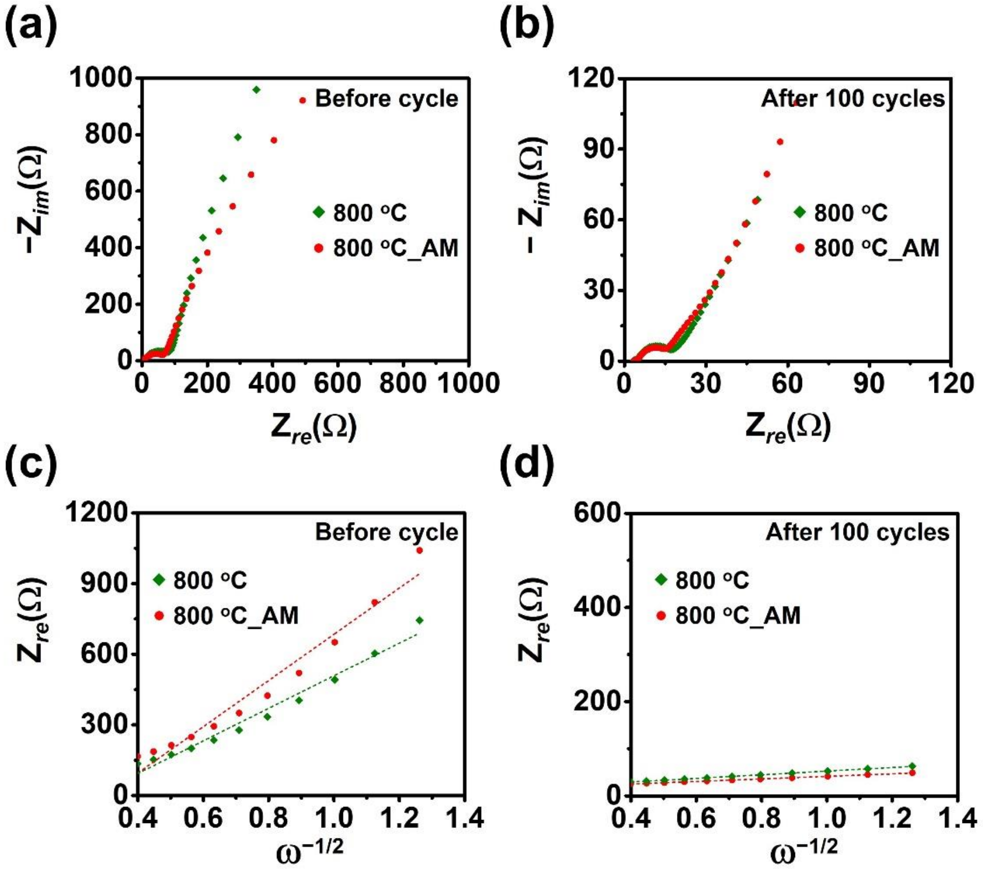

Figure S5. (**a**,**b**) Nyquist plots and (**c**,**d**) relationships between Z_{re} and $\omega^{-1/2}$ of the $ZnMn_2O_4$ powders post-treated at 800 °C temperatures and aggregated microsphere consisting of $ZnMn_2O_4$ nanoparticles (800 °C_AM): (**a**) before cycle, (**b**) after 100 cycles, (**c**) before cycle, and (**d**) after 100 cycles.

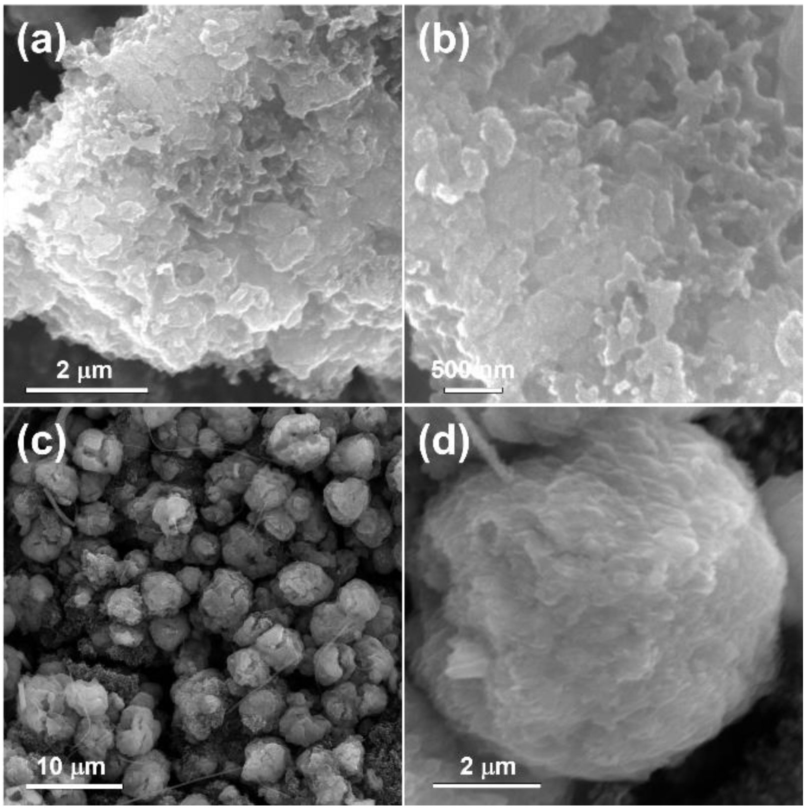

Figure S6. SEM images of (**a**,**b**) $ZnMn_2O_4$ powders post-treated at 800 °C temperatures and (**c**,**d**) aggregated microsphere consisting of $ZnMn_2O_4$ nanoparticles (800 °C_AM) after 100 cycles.

Table S1. Electrochemical properties with other $ZnMn_2O_4$ materials as anode materials for LIBs reported in the previous literatures.

**Materials**	**Current Rate**	**Discharge Capacity [mA h g^{-1}] and (Cycle Number)**	**Rate capacity [mA h g^{-1}] (Current Rate)**	**Ref**
$ZnMn_2O_4$ ball-in-ball hollow microspheres	0.4 A g^{-1}	750 (120)	396 (1.2 A g^{-1})	[1]
Pure phase of $ZnMn_2O_4$	0.2 A g^{-1}	458 (50)	205 (10.0 A g^{-1})	[2]
Porous $ZnMn_2O_4$ microspheres	0.5 A g^{-1}	800 (300)	400 (2.0 A g^{-1})	[3]
Hierarchical porous $ZnMn_2O_4$ hollow nanotubes	0.2 A g^{-1}	669 (280)	352 (2.0 A g^{-1})	[4]
$ZnMn_2O_4$ microspheres	0.1 A g^{-1}	602 (100)	483 (0.5 A g^{-1})	[5]
Porous $ZnMn_2O_4$ nanospheres	0.2 A g^{-1}	~800 (120)	300 (6.0 A g^{-1})	[6]
Porous $ZnMn_2O_4$ nanowires	0.5 A g^{-1}	869 (100)	345 (4.0 A g^{-1})	[7]
**Aggregated microsphere consisting of $ZnMn_2O_4$ nanoparticles**	**2.0 A g^{-1}**	**687 (100)**	**594 (4.0 A g^{-1})**	**Our work**

References

1. Zhang, G.; Yu, L.; Wu, H.; Hoster, H.; Lou, X.W.(. Formation of ZnMn2O4 Ball-in-Ball Hollow Microspheres as a High-Performance Anode for Lithium-Ion Batteries. *Adv. Mater.* **2012**, *24*, 4609–4613, https://doi.org/10.1002/adma.201201779.
2. Song, M. S.; Cho, Y. J.; Yoon, D. Y.; Nahm, S.; Oh, S. H.; Woo, K.; Ko, J. M.; Cho, W. I. Solvothermal synthesis of $ZnMn_2O4$ as an anode material in lithium ion battery. *Electrochimica Acta* **2014**, *137*, 266–272, https://doi.org/10.1016/j.electacta.2014.05.126.

3. Wang, N.; Ma, X.; Xu, H.; Chen, L.; Yue, J.; Niu, F.; Yang, J.; Qian, Y. Porous ZnMn2O4 microspheres as a promising anode material for advanced lithium-ion batteries. *Nano Energy* **2014**, *6*, 193–199, https://doi.org/10.1016/j.nanoen.2014.04.001.
4. Zhang, L.; Zhu, S.; Cao, H.; Hou, L.; Yuan, C. Hierarchical Porous ZnMn2O4Hollow Nanotubes with Enhanced Lithium Storage toward Lithium-Ion Batteries. *Chem. – A Eur. J.* **2015**, *21*, 10771–10777, https://doi.org/10.1002/chem.201501421.
5. Dang, W.; Wang, F.; Ding, Y.; Feng, C.; Guo, Z. Synthesis and electrochemical properties of ZnMn2O4 microspheres for lithium-ion battery application. *J. Alloy. Compd.* **2017**, *690*, 72–79, https://doi.org/10.1016/j.jallcom.2016.07.304.
6. Chen, X.; Zhang, Y.; Lin, H.; Xia, P.; Cai, X.; Li, X.; Li, X.; Li, W. Porous ZnMn2O4 nanospheres: Facile synthesis through microemulsion method and excellent performance as anode of lithium ion battery. *J. Power Sources* **2016**, *312*, 137–145, https://doi.org/10.1016/j.jpowsour.2016.02.056.
7. Zhang, Y.; Zhang, Y.; Guo, C.; Tang, B.; Wang, X.; Bai, Z. Porous ZnMn2O4 nanowires as an advanced anode material for lithium ion battery. *Electrochimica Acta* **2015**, *182*, 1140–1144, https://doi.org/10.1016/j.electacta.2015.10.032.
